# Finger Gesture Spotting from Long Sequences Based on Multi-Stream Recurrent Neural Networks

**DOI:** 10.3390/s20020528

**Published:** 2020-01-18

**Authors:** Gibran Benitez-Garcia, Muhammad Haris, Yoshiyuki Tsuda, Norimichi Ukita

**Affiliations:** 1 Toyota Technological Institute, Nagoya 468-8511, Japan; mharis@toyota-ti.ac.jp (M.H.); ukita@toyota-ti.ac.jp (N.U.); 2 DENSO CORPORATION, Kariya 448-8661, Japan; yoshiyuki.tsuda.j5f@jp.denso.com

**Keywords:** gesture spotting, human–computer interaction, automotive user interfaces, in-vehicle sensors, recurrent neural networks

## Abstract

Gesture spotting is an essential task for recognizing finger gestures used to control in-car touchless interfaces. Automated methods to achieve this task require to detect video segments where gestures are observed, to discard natural behaviors of users’ hands that may look as target gestures, and be able to work online. In this paper, we address these challenges with a recurrent neural architecture for online finger gesture spotting. We propose a multi-stream network merging hand and hand-location features, which help to discriminate target gestures from natural movements of the hand, since these may not happen in the same 3D spatial location. Our multi-stream recurrent neural network (RNN) recurrently learns semantic information, allowing to spot gestures online in long untrimmed video sequences. In order to validate our method, we collect a finger gesture dataset in an in-vehicle scenario of an autonomous car. 226 videos with more than 2100 continuous instances were captured with a depth sensor. On this dataset, our gesture spotting approach outperforms state-of-the-art methods with an improvement of about 10% and 15% of recall and precision, respectively. Furthermore, we demonstrated that by combining with an existing gesture classifier (a 3D Convolutional Neural Network), our proposal achieves better performance than previous hand gesture recognition methods.

## 1. Introduction

Gestures are a natural form of human communication [1]. Thus, gesture recognition presents an essential part of human–computer interaction (HCI). Systems using vision-based interaction and control are more common nowadays [2,3,4]. Compared to the traditional inputs such as mouse and keyboard, vision-based interfaces are more practical and natural to perform depending on the main task. In particular, in-vehicle interfaces controlled by hand and finger gestures have been implemented due to nonintrusive performance, and high-level user acceptability [5,6,7,8]. A more recent application involves HCI with a head-up display (HUD) in autonomous cars [9,10]. Full windshield HUDs can present useful driving and navigation information employing augmented reality [11], which can be manipulated by driver and passenger with a touchless interface using finger gestures. Automatic recognition of finger gestures is, therefore, the basis of such applications.

Finger gesture recognition can be divided into two stages: gesture spotting and gesture classification. Gesture spotting aims to detect temporal video segments that contain gesture instances, while the classification stage aims to classify the gesture of each spotted segment. The majority of gesture recognition methods focus on the second stage, assuming that only one gesture is included in each video sequence [12,13,14,15,16]. However, gesture spotting is needed for practical applications, since the duration and temporal boundaries of gestures are commonly unknown in practice [17,18]. It is worth noting that temporal action proposal generation (TAPG) is similar to gesture spotting, and receives more attention from the research community [19,20,21,22,23,24,25,26]. TAPG generates video segment *proposals* (candidates) that may contain human action instances from untrimmed videos. These candidate video segments are useful for action recognition, video recommendation, and video highlight detection [19,23].

**Figure 1:**
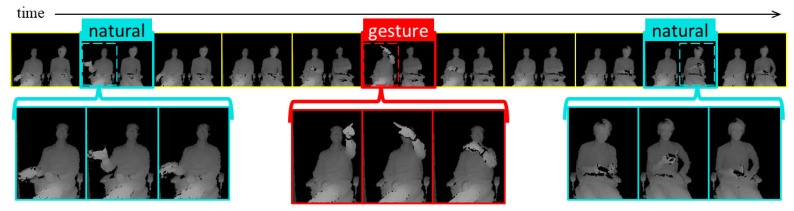
An example of the captured behavior of driver and passenger when interacting with an in-car touchless interface. “Natural” refers to natural behaviors of users’ hands, and “gesture” to target gestures. (**top**) A raw sequence from a depth video captured inside an autonomous car. (**left**) A spontaneous gesture of the driver when interacting with the passenger. (**center**) Example of a target gesture that triggers a command on the interface. (**right**) Interaction of the passenger with an object (smartphone).

Gesture spotting and TAPG methods share the same goal: to retrieve precise starting and ending temporal boundaries of segments that highly overlap with truth instances. However, many TAPG approaches are designed to work offline for specific applications, such as video recommendation and video highlight detection [19,22,23,24]. Furthermore, the TAPG ability to generate candidate video segments for unseen action categories (known as generalization ability) is praised due to its utility in video highlight detection. This ability arises because TAPG aims to detect the temporal segments when any human action appears. However, when controlling touchless in-car interfaces with finger gestures, the generalization ability of TAPG may spot undesired natural behaviors of users’ hands, which often take place in a real scenario, as shown in Figure 1. For example, spontaneous hand gestures may appear when driver and passenger interact in a regular conversation, as shown in the left part of Figure 1. Also, user interaction with objects may produce undesired detections, as shown in the right part of Figure 1. Some of these false-positive errors might be easily discarded in the classification stage (classified as non-target gestures). However, running a gesture classifier to many candidate video segments is computationally expensive. Therefore, a robust finger gesture spotting method should be able to reject the negative examples of Figure 1 (named natural), and to retrieve video segments which include only target gestures. In general, finger gesture spotting not only requires to retrieve precise temporal video segments, but also (1) overcome the similarities between gesture and non-gesture frames, (2) reject natural behaviors of users’ hands, and (3) be able to work online.

In this paper, we propose a finger gesture spotting method to overcome the mentioned difficulties. We based our proposed method in a state-of-the-art recurrent neural network (RNN) architecture designed for online TAPG [26]. We overcome the generalization ability of this baseline by merging hand and hand-location features obtained from a depth sensor, which help to discriminate target gestures from natural movements of the hand, since these may not happen in the same 3D spatial location. Our architecture builds upon the assumption that the discriminative ability increases if each branch on a multi-stream CNN specializes in specific cues [16,27]. Therefore, we include cues of hand and hand-location on a multi-stream RNN, which have been proven to improve the gesture classification performance [15,28]. Furthermore, hand-location features are extracted with a particular convolutional layer that gives access to its own input coordinates [29]. Thus, these features are specially designed to learn the hand location of target gestures.

**Figure 2:**
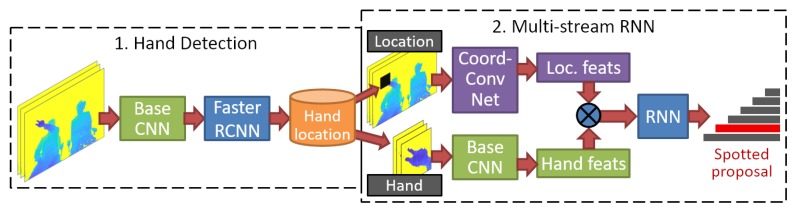
An overview of our approach. The input of the first module is a raw frame processed with faster R-CNN [30] to detect the hand that performs a finger gesture. In the first branch of the second module (highlighted in purple), hand-location features are extracted by a CoordConv network [29], which handles the input frame overlaid by a black mask on the detected hand region. In the second branch, the hand region is cropped, and features are extracted using the base CNN employed in the first module (highlighted in green). The combination of both features is feed to an recurrent neural network (RNN) architecture [26] trained to define the video segments that may contain a finger gesture. Finally, RNN outputs confidence scores of multiple candidates that finish at the current frame.

In detail, our approach is divided into two modules: hand detection and multi-stream RNN. The first module consists of detecting the hand that performs the finger gesture, as shown in the left part of Figure 2. In the second module, our proposed multi-stream RNN (right part of Figure 2) is based on an RNN network (the dark blue block) for spotting human actions online on long untrimmed video sequences [26]. This network is fed by hand and hand-location features extracted from depth images. For hand features, we crop the hand region and apply the same base network used for hand detection, as highlighted in green in Figure 2. Meanwhile, hand-location features are extracted with a CoordConv network [29] (purple block). Finally, the output of our network is the best-scored video segment candidate that finishes at the current frame. It is worth noting that the multi-stream CNN and RNN are jointly trained in a unified framework (multi-stream RNN).

To validate our method, we collect a finger gesture dataset in an in-vehicle scenario of an autonomous car. We captured 226 untrimmed videos with more than 2100 instances of continuous finger gestures with a depth sensor. Thus, our architecture can learn rich 3D spatial information of the user’s hands. We define six gestures designed to control a windshield HUD, which are natural to perform as a pointer.

In summary, the main contributions of this paper are:

- We introduce a finger gesture spotting method on long untrimmed videos for controlling full windshield HUD interfaces in autonomous cars. Specifically, we tackle the problem of discriminating target gestures from natural hand movements by proposing a multi-stream RNN architecture with specialized cues of hand and hand-location.
- We demonstrate that our approach outperforms state-of-the-art methods with high recall and high temporal precision. We also evaluate the errors of our method by presenting a statistical analysis, which shows the user behaviors that directly affect spotting performance.
- We verify that the precision of our spotted finger gestures provides a stronger basis for online gesture classification than prior methods. The integration with an existing gesture classifier leads to improved previous gesture recognition approaches.

## 2. Related Work

### 2.1. Temporal Action Proposal Generation

As aforementioned, the goal of TAPG is to generate temporal video segments (proposals) of human action instances, and most of the state-of-the-art approaches work offline [22,23,24]. These methods can work only offline because they need to process the whole video in order to retrieve high-quality candidates. However, this is not feasible for gesture recognition methods designed for online HCI, which needs to process the gesture immediately upon or even before their completion to provide fast feedback. On the other hand, there are TAPG methods that can detect actions on the fly. Among them, efficient convolutional network for online video understanding [21] can achieve action detection and recognition simultaneously by using a 3D CNN architecture based on temporal pooling of sliding windows. Besides, deep action proposals [25] and single-stream temporal approaches (SST) [26] introduce RNNs to generate video segments with only one single pass, avoiding the use of overlapped temporal sliding windows. Thanks to the recurrent process of RNN, SST retrieves the beginning of the instance finished on the latest evaluated frame of a long video sequence. This characteristic is essential for the online performance sought by HCI applications. However, one of the weakest points from RNN based methods is the hidden state saturation problem [31], which is caused if the input sequence is too long.

In addition, TAPG methods have a prominent generalization ability, which allows to generate temporal video segments for unseen action categories [22,23,24,25,26]. Even though this characteristic is beneficial for some applications, it produces undesired detections of natural behaviors of users’ hands for finger gesture spotting. In particular, our gesture spotting approach builds on the progress made by the SST architecture [26], and we propose to decrease the generalization problem by introducing a two-stream architecture that combines specialized streams of hand, and hand-location cues. In this way, the network can learn specific features that can help to differentiate between target gestures and natural hand movements, since these may perform in a slightly different 3D spatial location. Furthermore, we explicitly avoid the hidden state saturation problem by considerably reducing the input sequence. Specifically, our multi-stream RNN only process frames that present hand detections of target gestures. Hence, the hidden state is purged when no hands are detected for a certain number of frames.

### 2.2. Multi-Stream Gesture Recognition

Multi-stream architectures have been widely employed for action [20,22,23,32,33,34] and gesture recognition [12,13,14,15,16,27,35]. This technique consists of processing different versions of the same video in parallel with two or more CNNs. Karpathy et al. [32] were the first to propose a two-stream architecture for action recognition, which combines features learned from low-resolution frames (context stream) and high-resolution cues from the center of the frame (fovea stream). Simonyan et al. [33] were the pioneers of fusing features from two modalities, using one stream with RGB images and the other with flow fields. This multi-modality approach is prevalent for gesture recognition [12,13,14,15], as shown in the 2017 ChaLearn Look At People (LAP) gesture recognition challenge [36], where all the entries used multi-stream architectures of at least RGB and depth streams.

Recent studies have demonstrated that multi-stream architectures succeed if each stream specializes in specific cues [16,27]. In other words, an extra channel is only beneficial if it adds a new source of information, and the improvement is limited by the particular gestures that can be represented with these additional cues. Furthermore, the outputs of streams should be combined selectively, preferably through trained fusion networks as in [13,14,15,28,32,35]. For instance, Liu et al. [28] introduced a two-stream 3D-CNN combining hand-location features of RGB and depth modalities by explicitly overlaying a black mask on the input frames. Narayana et al. [15] combined 12 different channels, including modalities of RGB, depth, and flow for achieving state-of-the-art results of the isolated gesture detection (IsoGR) challenge of the ChaLearn LAP. Their results demonstrated that streams with specialized cues such as hand and hand-location contribute better than global channels that include information from the whole frame. Therefore, we use a two-stream architecture combining the hand and hand-location information, as shown in the right part of Figure 2. Based on this process, our approach can precisely learn the position of the hand, which is useful to exclude the natural behaviors of users’ hands with similar appearance than target gestures. A situation that often occurs when interacting with a windshield HUD in an autonomous car, as illustrated in Figure 1.

### 2.3. Gesture Spotting

Most of the previous methods consider the gesture spotting as the first step of gesture recognition. For instance, the winners [28] and the runner-ups [37] of the continuous gesture detection (ConGD) challenge of the 2017 ChaLearn LAP, spotted the gestures based on a dataset-specific observation: subjects raise their hands at the beginning of gestures and put them down again at the end. Similarly, Benitez-Garcia et al. [38] propose to detect temporal boundaries of the gestures using a triplet-loss network under the assumption that a general gesture starts and ends with a similar position and pose of the hand. One year after ConGD challenge, Zhu et al. [17] overcame the results of the winners by proposing a temporal dilated 3D CNN architecture to binary classify gesture/non-gesture frames. The current state-of-the-art method [18] uses the 12-channels architecture with extra RNN layers to simultaneously spot and classify continuous gestures. Recently, Kopuklu et al. [39] proposed the real-time hand gesture detection (RHGD) approach, which is based on a hierarchical structure of 3D CNN architectures to detect and classify continuous hand gestures. Their spotting method consists of detecting gesture frames using a shallow 3D-CNN model on eight consecutive frames.

The main drawbacks of the listed methods with respect to our intended application are twofold: (1) the freedom of the in-car scenario makes difficult to pre-define a data-specific property that can help the spotting, e.g., the position and pose of the hand can differ among the starting and ending temporal boundaries contradicting the assumptions of previous works [28,37,38]; (2) finger gestures usually share similarities between gesture and non-gesture frames, especially with natural behaviors of users’ hands, as illustrated in the left part of Figure 1. An issue that has not been considered by previous works. Therefore, in this paper, we propose a method that includes a multi-stream CNN based on specialized streams of hand and hand-location cues, which combined with an RNN can spot finger gestures without pre-defining data-specific restrictions.

## 3. Proposed Method

As illustrated in Figure 2, our approach is divided into two modules: hand detection and multi-stream RNN. In the resume, the first module focuses on the detection of the hand that performs the finger gesture. Subsequently, the cropped hand region and the hand-location are feed to the multi-stream RNN. Note that the base network of hand detection is also employed in the hand features stream (green colored conv. block). Thus, the trainable parameters in the second module are reduced. Finally, RNN outputs the best scored temporal proposal that finishes at the current frame. In this section, we introduce the technical details of each module and describe the training process, which helps to mitigate the inherited problems related to RNNs at inference time.

### 3.1. Hand Detection

**Figure 3:**
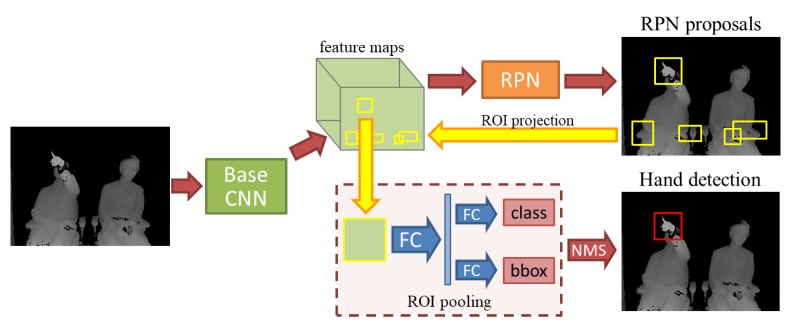
Hand detection process based on faster RCNN [30]. The raw frame is processed by the base CNN, which in our case comprises the first convolutional layers of ResNet-101 [40]. The region proposal network (RPN) generates regions of interest (ROI) from the feature maps obtained by the base CNN. Subsequently, ROIs are pooled and mapped to feature vectors by fully connected layers (FC), which are used for classification and regression of bounding boxes. Finally, the hand region is defined by the non-maximum suppression (NMS) algorithm.

Hand detection of the target gesture is crucial for our proposal, since the input frame may include visual information from both driver and passenger of the autonomous car. Therefore, we employ the well-known object detection algorithm of Faster R-CNN [30] to build our hand detection model. The process used for Faster R-CNN is illustrated in Figure 3. The region proposal network (RPN) generates high-quality regions of interest (ROI) which may contain hand-shaped objects, as shown in the upper branch of Figure 3. Subsequently, all ROIs are pooled and mapped to feature vectors by fully connected layers (FC) used for classification and regression of bounding boxes. Finally, non-maximum suppression (NMS) is performed to define the final hand region, as shown in the bottom branch of Figure 3.

### 3.2. Multi-Stream RNN

**Figure 4:**
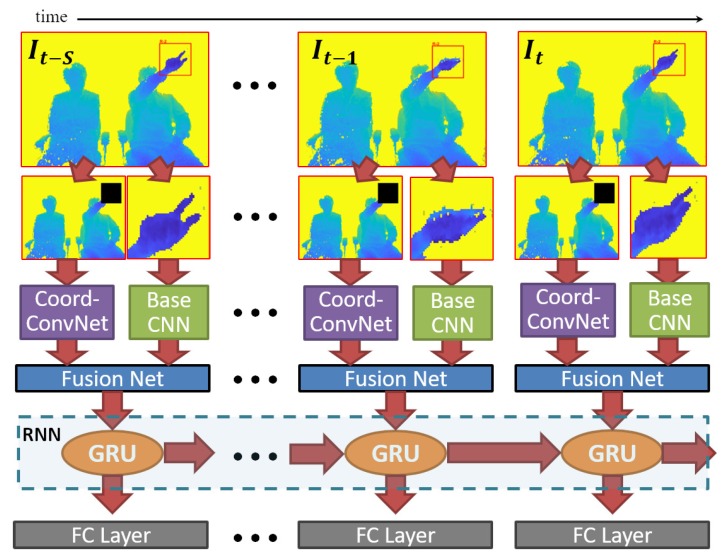
The frame-wise process of our multi-stream RNN. In the first stream, the input frame is overlaid by a black mask in the hand location and processed by a CoordConv network (purple colored block). On the second stream, the hand region is cropped and processed by the base CNN (green colored block). The fusion network combines both types of features for feeding the RNN with GRU cells. Finally, a fully connected layer outputs confidence scores of possible proposals ending at the current frame.

Figure 4 illustrates the process of our multi-stream RNN method. Let $I_{t}$ be the current frame and also the ending boundary of a finger gesture. The gesture consists of a sequence of S+1 frames $\left\{I_{t-S},\ldots ,I_{t-1},I_{t}\right\}$, where $I_{t-S}$ represents the starting boundary frame. We propose to combine specialized streams of hand and hand-location cues (extracted from each $I_{i}$ input frame) with a fusion network. The fused features are fed to an RNN which recurrently learns semantic information with gated recurrent unit (GRU) cells. Finally, the output confidence scores of *k* possible proposals are given by a fully connected layer which takes the hidden state embedding of the GRU layer as an input. In this subsection, we describe each step involved in our multi-stream architecture.

Hand-location features are extracted with a CoordConv network (highlighted in purple in Figure 4), which works by adding extra coordinate channels to the input image. In this way, the learning process allows convolutional access to the input Cartesian space [29]. Thus, two channels are added in the first convolutional layer, regarding *X* and *Y* spatial position, while the rest of the network remains unchanged. We build a CoordConv net with 7 × 7 convolutional filters in the first layer, followed by three bottleneck blocks (defined in [40] for ResNet-50/101/152). Table 1 shows details of the CoorConv net architecture, including the number of trainable parameters.

Hand features are extracted with part of the base network used in the previous module. Rather than using all the convolutional layers of ResNet-101 [40] (as in hand detection module), the base network for hand features comprises only the first two layers, as described in Table 1. Note that the number of trainable parameters of this network is zero because we share the weights learned in the hand detection process.

The fusion network is proposed at an intermediate level because it has been demonstrated that optimal performance is achieved when multi-stream features are combined selectively through trained fusion networks in mid or late levels [16,27]. We concatenate the feature maps of hand and hand-location streams to feed our fusion network. The architecture of this network consists of two ResNet blocks [40], similar to the building blocks of ResNet-18. The output of the last convolutional layer is pooled, and the resultant 512-D feature vector is processed by the RNN. Table 1 shows further details of the fusion net architecture.

RNN with GRU. Following SST [26], we adopt an RNN architecture able to process videos of an arbitrary length without needing to employ overlapping temporal windows. The RNN builds upon GRU instead of long short-term memory (LSTM) cells for sequence encoding, which results in a slightly better performance with fewer parameters, as demonstrated in previous evaluations [31,41].

GRU was proposed to make each recurrent unit to adaptively capture dependencies of different time scales [41]. Hence, the activation $h_{t}$ of the GRU at time *t* is a linear interpolation between the previous activation $h_{t-1}$ and the candidate activation $\tilde{h_{t}}$. The formulation for defining $h_{t}$ of the GRU is as follows:

(1) $$\begin{array}{c} h_{t}=\left(1-z_{t}\right)⊙h_{t-1}+z_{t}⊙\tilde{h_{t}}\\ \tilde{h_{t}}=\tanh\left(Wx_{t}+r_{t}⊙\left(Uh_{t-1}\right)+b\right), \end{array}$$

where ⊙ is the Hadamard product, $z_{t}$ represents the update gate, $x_{t}$ is the output of our fusion network, and $r_{t}$ represent the reset gate at time *t*, while *W*, *U* and *b* are learnable weights and bias, respectively. The update gate *z* decides how much the unit updates its activation, or content, while the reset gate *r* makes the unit act as if it is reading the first frame of an input sequence, allowing it to forget the previously computed state. Both gates are similarly computed:

(2) $$\begin{array}{c} r_{t}=\sigma_{r}\left(W_{r}x_{t}+U_{r}h_{t-1}+b_{r}\right)\\ z_{t}=\sigma_{z}\left(W_{z}x_{t}+U_{z}h_{t-1}+b_{z}\right), \end{array}$$

where $x_{i}$ and the previous activation $h_{i-t}$ are multiplied by its own weights *W* and *U*, respectively, while σ represents a logistic sigmoid function.

Finally, at each time step *t*, a fully conected layer with softmax produces confidence scores corresponding to *k* proposals $P_{t}={\left\{\left(p_{t-j},p_{t}\right)\right\}}_{j=1}^{k}$, where $\left(p_{t-j},p_{t}\right)$ represents a proposal which starts at $p_{t-j}$ and ends at $p_{t}$, as defined by [26]. In this way, the final decision of the RNN is based on the argmax of *k* confidence scores greater than a detection threshold Th.

### 3.3. Training Process

The hand detection module is trained using hand regions included in sequences of target gestures only. Thus, the network is forced to learn hand poses that must be included in gestures that we want to classify. In this way, detections of hands depicting no target gestures may be avoided. On the other hand, the training of the multi-stream RNN is more complex and should be carefully designed, since it has to provide proper information to learn the behavior of long videos while avoiding overfitting.

The original training procedure of SST [26] was designed to fully unroll the RNN over very long input sequences at test time. The process is based on densely sampled, overlapped training video segments of size *L* that is significantly longer than the temporal proposals to be detected. However, there are two main drawbacks to this process: (i) the densely sampled training videos generate a huge imbalance among positive and negative samples, and (ii) at inference time, the RNN still has to manage very long input sequence, which may saturate its hidden state. Therefore, we address these issues employing detections generated in the first module. We mitigate difficulty (i) by an adaptive sampling of sequences with hands detected, which densely sample videos that overlap with target gestures, and sparsely sample the rest of the segment. In this way, we focus the training only on the most relevant parts of the videos. Equally important, we solve the problem (ii) by explicitly clearing the hidden state when δ consecutive no-gesture hands are detected (we experimentally set δ=5 frames).

**Figure 5:**
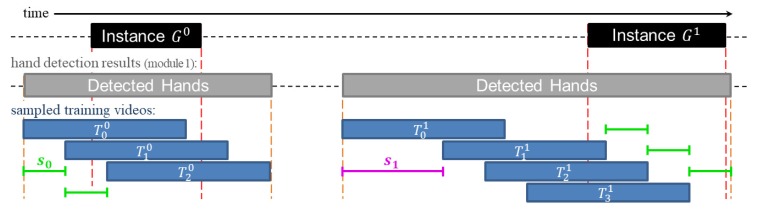
A representation of the adaptive sampling of training videos. (**left**) Training videos that overlap with a truth instance are sampled with stride $s_{0}$. (**right**) Training videos that do not overlap with any truth instance are sampled with stride $s_{1}$.

We generate training samples using all continuous sequence of frames where hands were detected, as illustrated in Figure 5. Training sequences *T* that do not overlap with temporal intervals of any target gesture ${\left\{G^{j}\right\}}_{j=0}^{q}$ are sampled with a higher stride than those who do overlap. For example, the first target gesture $G^{0}$ of Figure 5 is sampled on three segments using stride $s_{0}$ because any possible sequence $T^{0}$ of size *L* will overlap with $G^{0}$. However, the second instance $G^{1}$ may include training samples that do not overlap with the target gesture. Hence, these are sampled with a higher stride $s_{1}$, so that $s_{1}>>s_{0}$.

Similar to [26], each training sequence is associated with ground truth labels that indicate which time intervals correspond to the target gesture. For example, $T_{0}^{0}$ from Figure 5 will be associated with a set of labels $Y_{0}^{0}={\left\{y_{l}\right\}}_{l=1}^{L}$, where the label $y_{l}$ is a *k* dimensional binary vector. So that, the *j*-th element of ${\left\{y_{l}^{j}\right\}}_{j=0}^{k}$ is set to 1 if its corresponding proposal interval has a temporal intersection-over-union (tIoU) with the $G^{0}$ larger than 0.8, and set to 0 otherwise. Finally, the multi-stream RNN is penalized for errors according to a weighted binary cross-entropy loss function.

## 4. Experimental Results

### 4.1. Dataset

**Figure 6:**
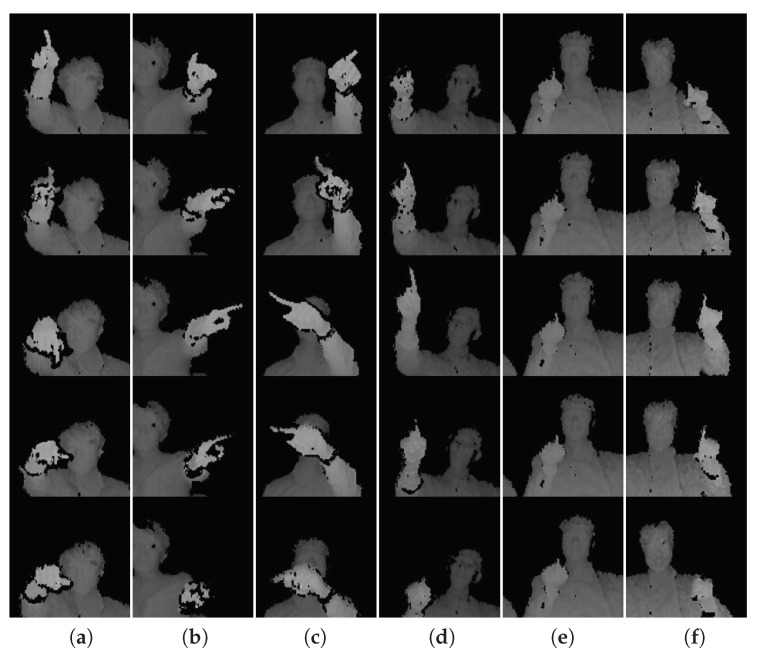
Example of the six gestures of our dataset. The temporal order of each gesture progresses from the top to bottom rows. (**a**) Flicking-down, (**b**) flicking-left, (**c**) flicking-right, (**d**) flicking-up, (**e**) pointing, and (**f**) pushing. Note that for better visualization, frames with zoom-in are shown. Refer to the Figure 1 for an example of raw frames.

We evaluate our gesture spotting approach with a new dataset, which consists of 226 untrimmed depth videos with 2166 instances of continuous finger gestures. Each video sequence comprises more than 5400 frames (3 min. of duration) and contains about ten gesture instances. The average duration of a hand gesture is 52 frames (less than two seconds). As shown in Figure 6, we define six gestures natural to perform in an in-vehicle scenario: flicking-down (F.Do.), flicking-left (F.Le.), flicking-right (F.Ri.), flicking-up (F.Up.), pointing (Po.), and pushing (Pu.).

We collect the data simulating an in-vehicle scenario of an autonomous car, where driver and passenger performed gestures for controlling a full windshield HUD. The data collection setup includes an ultra-short-throw projector, projected on a white screen to simulate a windshield HUD. The depth camera is virtually located in the center dashboard between passenger and driver seats, with a distance of 140 cm between the camera and the backrest of the seats. A total of 12 subjects participated in at least two sessions for capturing the data. The subjects were instructed to act naturally as in an autonomous car, and performing a valid gesture pointing to a position mark that randomly appears on the screen. The position mark overlays the driver’s view, which is an urban street scene. We use an ASUS Xtion2 (TOF) camera with a depth sensor (acquired from ASUSTeK COMPUTER IN, Taipei, Taiwan) to record video clips at 30 fps. Individual frames of the video sequences were normalized to 640 × 480 pixels, 8-bit depth.

The whole dataset was randomly divided into training, validation, and testing sets, comprising 1378, 277, and 511 instances, respectively. The amount of instances for each considered gesture is detailed in Table 2. Bounding boxes of target gestures (only the hand which is performing the action) as well as temporal boundaries of all instances were manually annotated. Figure 4 shows some examples of the annotated bounding boxes.

### 4.2. Implementation Details

All experiments were conducted using Python 3.5.2 and PyTorch 1.0 on a single NVIDIA TITAN X GPU. For the hand detection module, we input the raw frames with its original size, and all training parameters are defined as in [30]. For multi-stream RNN, we resized the input frame and the cropped hand to 160 × 160 pixels. We vary the length of training videos *L* and possible *k* proposals, finding the best combination as demonstrated in the following sub-section. We optimize the end-to-end multi-stream RNN with backpropagation using the SGD update with an initial learning rate of 0.01, and decreased every 15 epochs by factor 10.

### 4.3. Ablation Studies

Results for the hand detection module are evaluated using mean average precision (mAP), a conventional measure for object detection accuracy [42]. The mAP score is calculated by taking the average value of the precision across all recall values using an IoU threshold of 0.5. To evaluate the ratio of possible missed hand detections, we also measure the average recall score of each sub-set. Table 3 shows the results of hand detection, we observe that Faster R-CNN can achieve very high results for detecting target hands using depth images.

We evaluate the gesture spotting performance based on the average recall (AR) score, which is widely employed for evaluating TAPG [22,23,24,25,26]. The AR score is calculated by taking the average recall of the temporal Intersection-over-Union (tIoU) over all gesture classes with a certain tIoU threshold. The tIoU is an intersection over union measure that evaluates the predicted starting and ending points of the gesture. Thus, the tIoU score counts when the IoU between predicted and ground-truth temporal boundaries (starting and ending points) is higher than the tIoU threshold.

Baseline. We consider a single stream RNN extracting features from full-frames as a baseline. Note that this method also follows the architecture and training described in Table 1 and Section 3.3, respectively. Thus, our baseline is defined to solve a gesture spotting problem rather than the original SST [26] proposed for TAPG.

**Figure 7:**
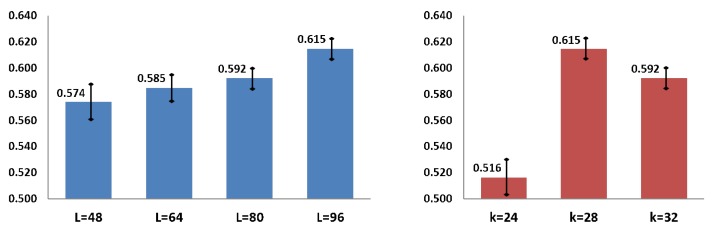
Validation results of the baseline method with different hyperparameter values. *Y*-axis shows the average recall (AR) score. (**left**) Results varying the training sequence length *L*. (**right**) Results varying the number of proposals *k*.

Evaluation of the hyper-parameters. We first evaluate the results of the baseline method by varying the hyper-parameters *L* and *k*. The results are shown in Figure 7, we observe that the highest performance is obtained with L=96 and k=28. These results follow the findings of the original SST [26], which demonstrates that better performances are obtained when the training sequence length is significantly longer than the number of proposals (L>>k). Note that with k=28 we cover the 95% of all gestures length from our dataset.

**Figure 8:**
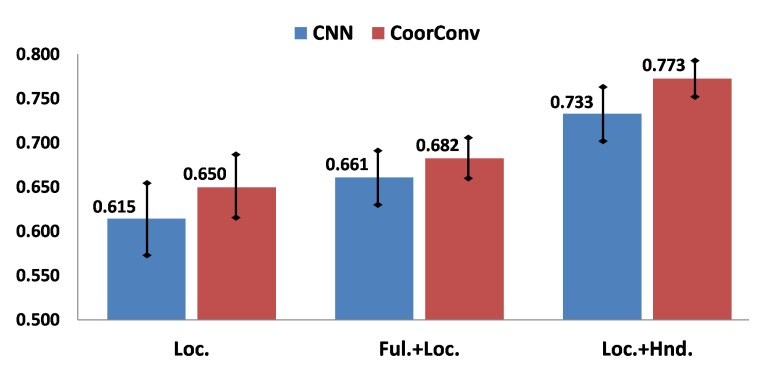
Validation results of multi-stream combinations that includes hand-location features with and without CoorConv network. *Y*-axis shows the AR score.

CoordConv Contribution. We evaluate the contribution of the CoordConv network [29] for extracting the hand-location features. Results of Figure 8 were obtained with all two-stream combinations that include hand-location features. Loc., Hnd., and Ful. refer to hand-location, hand, and full-frame features, respectively. All the results of CoordConv network outperform those from conventional CNNs, which demonstrates that adding extra coordinate channels to the input image contributes to better defining the hand location. Thus, this may lead to better discrimination ability between target gestures and natural hand movements. Furthermore, the combination of Loc. + Hnd. features presents a higher AR score than the Ful. + Hnd. combination, with about 10% of improvement.

**Figure 9:**
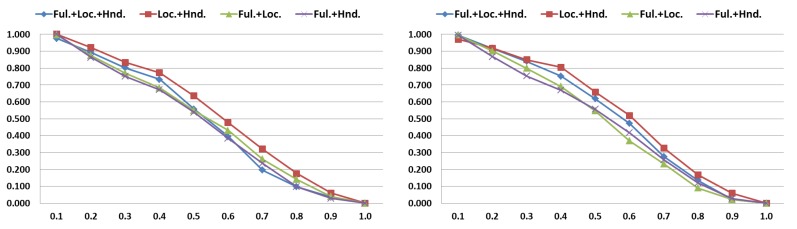
AR results of all feature combinations (hand-location, hand, and full-frame) for our multi-stream RNN. *X*-axis defines the different temporal intersection-over-union (tIoU) thresholds. (**left**) Validation results. (**right**) Testing results.

Evaluation of the multi-stream RNN. We evaluate our multi-stream architecture with different feature combinations, including hand-location, hand, and full-frame features. AR results with different tIoU are shown in Figure 9. We observe that the combination of hand-location and hand features (Loc.+Hnd. plotted in red on both graphs) achieves the best accuracy. This approach even overcomes the results of the combination of all cues (Ful. + Loc. + Hnd.), demonstrating that the combination of specialized streams contributes more than global features extracted from full-frames, as pointed out by previous works [15,28]. The similar accuracy of other combinations suggests that only Loc. and Hnd. features specialize in specific rich information, while the rest of the combinations may include redundant information, such as in Ful. + Hnd. which in general presents the lowest accuracy. From now on, when we mention the multi-stream approach, we refer to the two-stream RNN which merges hand-location and hand features (Loc. + Hnd.).

### 4.4. Comparison with Previous Works

**Figure 10:**
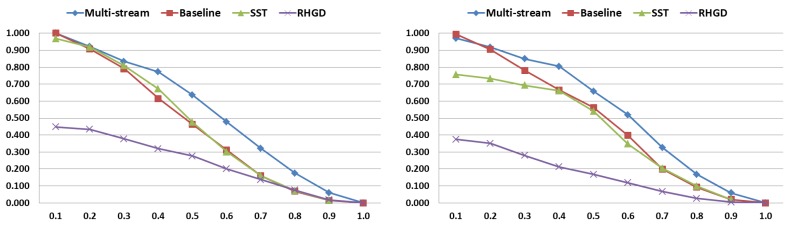
Results comparison of our approach with previous methods, such as SST [26] and real-time hand gesture detection (RHGD) [39]. *X*-axis defines the different tIoU thresholds, and *y*-axis the AR score. (**left**) Validation results. (**right**) Testing results.

We compare the performance of our approach with the defined baseline, the original SST, and the recent RHGD method. The SST was implemented with the same hyper-parameters as our approach (optimized for hand gestures rather than human actions). For RHGD [39], we use their publicly available code with the default hyper-parameters. The results of these methods with different tIoU thresholds are shown in Figure 10. We observe that our approach, in general, outperforms the previous works. Results of baseline and SST are similar even though the training procedure is different, which demonstrates that the main improvement is due to our multi-stream approach rather than the optimization of hyper-parameters. On the other hand, the low results of RHGD might be related to the input type (only depth images) and problems to process full-frames that may include visual information from more than one subject (frames including four hands in the worst case).

**Figure 11:**
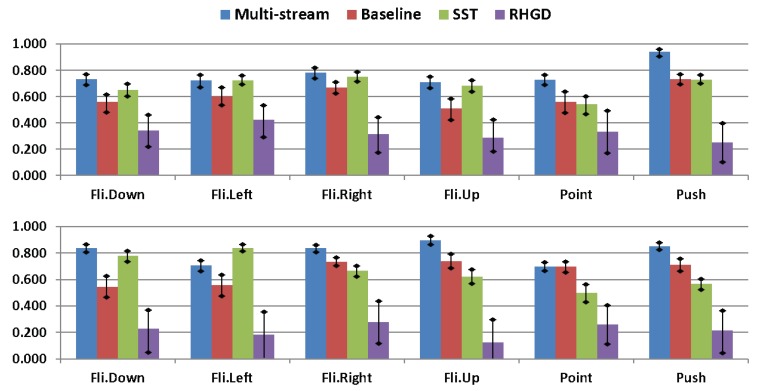
Comparision of AR results per class. (**top**) Validation results. (**bottom**) Testing results.

Figure 11 illustrates the gesture spotting results per-class from each evaluated method. As expected, our multi-stream RNN approach outperforms previous works in almost all gestures. Interestingly, we achieve a balanced AR results for each class (0.70–0.80), except for the pushing gesture which presents a higher score. This result might be related to the apparent 3D position difference through time shown by the hand when performing this gesture, information captured by depth features focused on the hand location.

### 4.5. Statistical Analysis of Errors

**Figure 12:**
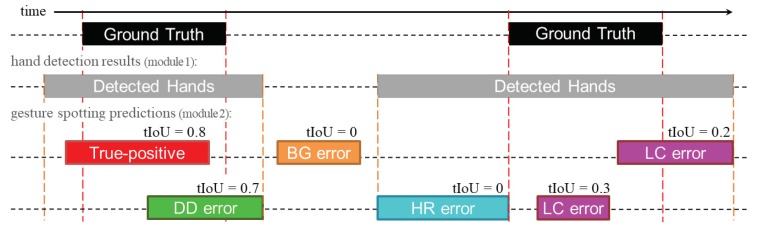
Examples of different types of false-positive errors: double-detection (DD), location (LC), hand-detection (HD), and background (BG). Note that the tIoU of each prediction is calculated with respect to their nearest ground-truth.

To better understand the false-positive (FP) errors of our gesture spotting approach, we present a statistical analysis. Following the findings of Alwassel et al. [43], our analysis considers four different error types: double-detection errors (DD) include predictions that were not the first to be assigned to a ground-truth (GT); location errors (LC) represent predictions that have tIoU >0 but do not satisfy the tIoU threshold; hand-detection related errors (HR) are predictions that have tIoU ≤0 but do overlap with continuous hand detections (achieved by module 1); and background errors (BG) include predictions that do not overlap with any GT nor hand detections. As shown in Figure 12, each error type is based on their temporal location with respect to the ground-truth, and/or with respect to the continuous hand detections achieved by the hand detection module. In the example of Figure 12, five FP errors are detected, while only one detection is correct (true-positive). So, the recall and precision scores are 0.5 and 0.16, respectively.

Table 4 presents the analysis of the four different errors of our multi-stream approach compared with the original SST method. Our proposed method presents higher precision and significantly fewer FP errors than SST. Furthermore, our approach completely eradicates BG errors and presents higher accuracy by decreasing the DD errors.

**Figure 13:**
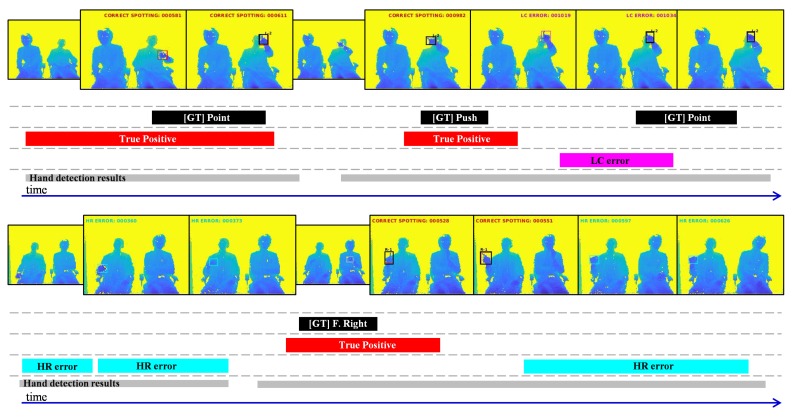
Qualitative results of our multi-stream approach. (**top**) A video sequence including pointing and pushing gestures. (**bottom**) A video sequence including a flicking-right gesture. The color-coded bars show the temporal location of the ground-truth and predictions (true-positives and false-positive), as well as the continuous hand detections achieved by module 1.

In Table 5, we also analyze the cause of the HR and LC errors of our approach. After manually analyzing all concerned predictions, we identify four main factors for the errors: (1) abrupt natural behaviors of users’ hands; (2) contiguous hand motions (before and after the target gesture); (3) pointing-related detections (target gesture segmented by erroneously detecting a pointing gesture); and (4) users’ interactions with objects. From Table 5, we observe that the problems are mainly related to the natural movements of the hands (as illustrated in Figure 1) as well as, the contiguous hand motions, which are the leading cause of LC errors. Figure 13 shows qualitative results of our multi-stream approach, including the most recurrent problems. For example, the bottom video sequence shows a subject that places his hand in a similar position than the pointing gesture. Thus, this situation leads to HR errors.

### 4.6. Gesture Recognition with Our Proposals

We evaluate the gesture recognition performance using our temporal spotted proposals. We employ the gesture recognition method of [39], which employs a 3D-CNN architecture based on ResNext-101 [44]. We train the gesture recognition using only ground-truth gesture sequences (without temporal results from hand-detections), while for inference, we use all temporal predictions obtained from the gesture spotting method. With this unique trained model, we test the temporal spotted proposals from our multi-stream approach, as well as those from the works described in the previous sub-sections.

Table 6 presents recall, precision, and f1 scores of each method. Notably, multi-stream results are substantially better than previous works. In particular, our method outperforms the recall of the baseline method by 8% and 14% with validation and test sets, respectively. In addition, we observe that our approach is considerably better than the baseline method in the testing set, with more than 23% of f1 score improvement. Furthermore, we significantly improve the recall of SST and RHGD, which demonstrates that the quality of our temporal proposals helps to improve the final gesture recognition performance.

Table 6 also presents the inference time of the complete process (spotting and recognition) and the spotting process only (shown in parenthesis) of each evaluated method. The only real-time approach is RHGD, but its accuracy is notably lower than our method. It is worth noting that the hand detection step of our approach consumes more than 65% of the total inference time. Therefore, we might reach real-time performance by simply replacing the backbone (ResNet-101) with a more efficient real-time network, such as HarDNet [45].

Finally, in Table 7, we present confusion matrices of our approach applied to validation and testing sets. As expected, the gestures with the highest recognition accuracy are Flicking-Left and Flicking-Right. On the other hand, the most misrecognized gesture is Flicking-Up. Interestingly, Pushing was the best-spotted gesture (as illustrated in Figure 11) but achieves the second-lowest recognition accuracy. This might be related to the apparent similarities to different classes such as pointing and flicking-up (as shown in the confusion matrices).

## 5. Conclusions

In this paper, we introduced a multi-stream recurrent neural network for finger gesture spotting, which works online and overcomes the similarities between gesture and non-gesture hand poses. Our multi-stream RNN network combines hand and hand-location features, which are located with a hand detection module applied to depth images. We have demonstrated that this combination helps to discriminate target gestures from natural hand movements, since these may not happen in the same spatial location. Extensive experiments on a collected finger gesture dataset of an in-vehicle scenario validate the effectiveness of our proposed architecture. Future work includes an end-to-end optimization of the hand detection and multi-stream RNN modules, as well as extending this framework to include the automatic gesture recognition of spotted temporal regions.

## Figures and Tables

**Table 1:** Architecture details of the three CNN networks used in our multi-stream RNN. *C*, *F*, *S*, and *N* correspond to the # of input channels, # of filters, stride, and # of building blocks in the layer. The output size of feature maps is based on the input frame resized to 160 × 160 pixels.

Layer	Output	CoorConv Net	Base Net (Hand)	Fusion Net
conv1	80 × 80	Conv. 7 × 7, C=3, F=64, S=2	Conv. 7 × 7, C=1, F=64, S=2	-
conv2	40 × 40	C=64, F=256, S=2, N=3	C=64, F=256, S=2, N=3	-
conv3	20 × 20	-	-	C=512, F=256, S=2, N=2
conv4	10 × 10	-	-	C=256, F=512, S=2, N=2
pool	1 × 1	-	-	average pooling
# trainable parameters		$0.21\times 10^{6}$	0	$11.4\times 10^{6}$

**Table 2:** The split of the dataset used in this paper.

Set	Fli.Down	Fli.Left	Fli.Right	Fli.Up	Point	Push	Total
Training	220	220	220	220	249	249	1378
Validation	44	44	44	44	51	50	277
Testing	80	80	80	80	96	95	511

**Table 3:** Hand detection results.

Training		Validation		Testing	
***recall***	***mAP***	***recall***	***mAP***	***recall***	***mAP***
1.000	0.999	0.999	0.997	0.994	0.974

**Table 4:** Analysis of multi-stream and SST results based on the four different types of false-positive (FP) errors, as described in Figure 12.

Method	Subset	Recall	Precision	FP	DD Err.	LC Err.	HR Err.	BG Err.
SST	valid	0.671	0.227	627	99	80	401	47
Multi-stream	valid	0.773	0.384	344	56	56	232	0
SST	test	0.660	0.177	1227	178	149	640	260
Multi-stream	test	0.804	0.503	389	54	55	280	0

**Table 5:** Analysis of error cause of LC and HR of our multi-stream approach.

	Validation			Testing	
**Error Cause**	**LC Err.**	**HR Err.**		**LC Err.**	**HR Err.**
Natural behavior	29%	60%		39%	66%
Contiguous motions	51%	10%		41%	1%
Pointing pose	20%	10%		20%	14%
Object interaction	0%	20%		0%	19%

**Table 6:** Detailed comparison of gesture recognition results based on temporal proposals of our multi-stream approach and previous works.

	Validation			Testing			Inference
**Method**	**Recall**	**Precision**	**f1-Score**	**Recall**	**Precision**	**f1-Score**	**Time**
RHGD	0.303	0.798	0.439	0.22	0.688	0.333	41 (315) fps
SST	0.513	0.634	0.567	0.47	0.696	0.561	22 (40) fps
Baseline	0.633	0.756	0.689	0.525	0.634	0.574	4.9 (5.5) fps
Multi-stream	0.719	0.797	0.756	0.662	0.752	0.704	4.3 (4.8) fps

**Table 7:** Confusion matrices of our multi-stream approach. Rows represent the accuracy (%) of the predictions.

	Validation						Testing					
	**F. Do.**	**F. Le.**	**F. Ri.**	**F. Up.**	**Po.**	**Pu.**	**F. Do.**	**F. Le.**	**F. Ri.**	**F. Up.**	**Po.**	**Pu.**
**F. Do.**	**76**	0	0	15	2	7	**86**	0	1	9	0	4
**F. Le.**	3	**93**	0	3	0	3	4	**85**	1	4	1	4
**F. Ri.**	5	5	**83**	0	5	2	0	0	**92**	3	0	5
**F. Up.**	0	0	0	**39**	47	13	22	0	0	**52**	21	4
**Po.**	5	2	0	7	**76**	11	6	1	3	13	**64**	13
**Pu.**	0	2	5	10	8	**75**	8	0	4	16	16	**55**
